# Paraneoplastic Pemphigus Presenting as Mild Cutaneous Features of Pemphigus Foliaceus and Lichenoid Stomatitis with Antidesmoglein 1 Antibodies

**DOI:** 10.1155/2010/931340

**Published:** 2010-07-12

**Authors:** Yayoi Niimi, Bungo Ohyama, Giovanni Di Zenzo, Valentina Calabresi, Takashi Hashimoto, Seiji Kawana

**Affiliations:** ^1^Department of Dermatology, Nippon Medical School, 1-1-5, Sendagi, Bunkyo-ku, Tokyo 113-8603, Japan; ^2^Department of Dermatology, Kurume University of Medicine, Kurume 830-0011, Japan; ^3^Molecular and Cell Biology Laboratory, IDI-IRCCS, 10400167 Rome, Italy

## Abstract

Herein, we report a case of paraneoplastic pemphigus with mild skin features of pemphigus foliaceus and lichenoid stomatitis associated with B-cell lymphoma. A 49-year-old man presented with scattered blisters and erosions on the trunk along with mucosal blisters and erosions. Skin biopsy showed subcorneal acantholytic bulla and oral mucosal biopsy demonstrated lichenoid dermatitis. Direct immunofluorescence showed cell surface deposits of IgG and C3. Indirect immunofluorescence identified circulating IgG autoantibodies to the cell surfaces of normal human skin and also on the transitional epithelium of rat bladder. Enzyme-linked immunosorbent assay using recombinant baculoproteins showed positive antidesmoglein 1 autoantibodies (index 46) but negative antidesmoglein 3 autoantibodies (index 8). Immunoblot analysis using normal human epidermal extract detected BP230 and the 190 kDa periplakin, while immunoprecipitation using radiolabeled cultured keratinocyte immunoprecipitated BP230 and the 210 kDa envoplakin. We consider that the skin lesion was produced by humoral immunity whereas the oral lesion was produced by cellular immunity.

## 1. Introduction

 Paraneoplastic pemphigus (PNP) was first reported in 1990 by Anhalt et al. as an autoimmune blistering disorder associated mostly with lymphoproliferative malignancies with characteristic clinical presentation and immunological findings [1, 2]. Recently PNP has been recognized as a wide spectrum of clinical and histological diseases with variable autoantibody profile involving both humoral and cellular autoimmunity response [1]. We report a case of PNP with cutaneous features of pemphigus foliaceus (PF) and lichenoid stomatitis and antibodies that were reactive with rat bladder epithelium. Interestingly, enzyme-linked immunosorbent assay (ELISA) detected only antidesmoglein 1 (Dsg1) antibodies but not anti-Dsg3 antibodies. The results of immunoblotting and immunoprecipitation were complex.

## 2. Case Report

**Figure 1:**
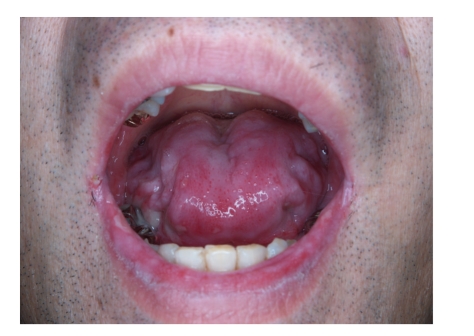
Blisters and erosions on the buccal mucosa, tongue, and lips. Oral lesions did not extend onto the vermillion of the lips.

**Figure 2:**
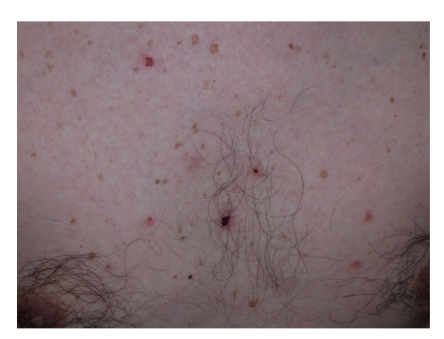
Scattered crusted and erosive lesions with flaccid bullae on the chest.

**Figure 3:**
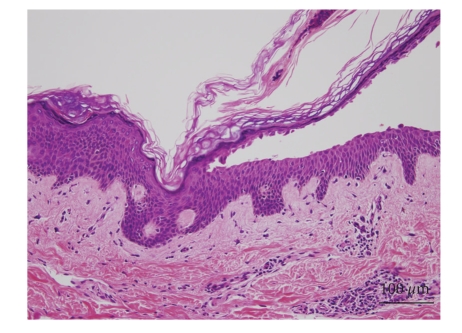
Histological findings of the skin lesion. Subcorneal bulla with mild acantholysis. No interface dermatitis was seen.

**Figure 4:**
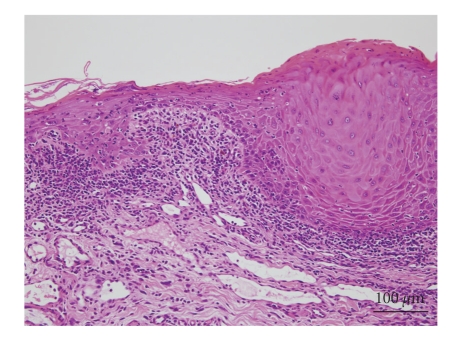
Histological findings of the oral lesion. Severe lichenoid dermatitis with dyskeratotic cells.

**Figure 5:**
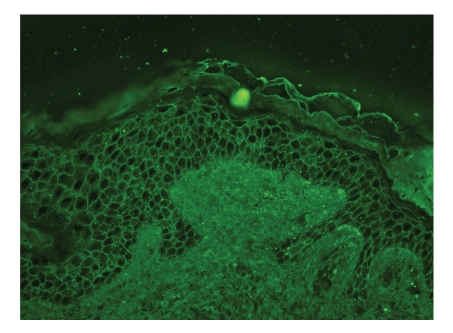
Direct immunofluorescence of the perilesional skin. Intercellular deposits of IgG throughout epidermis.

**Figure 6:**
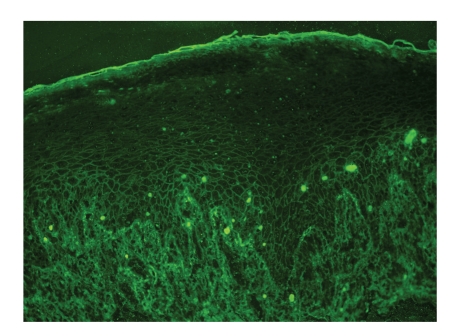
Direct immunofluorescence of the oral mucosa. Cell surface deposits of IgG in the lower layer of epithelium with ovoid bodies.

**Figure 7:**
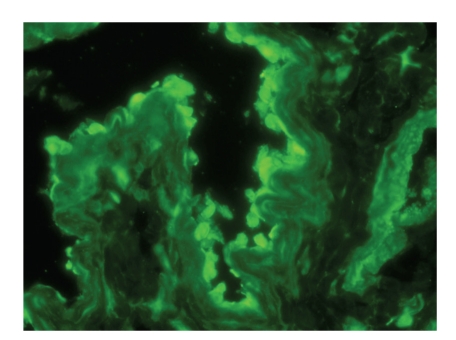
Indirect immunofluorescence was positive using rat bladder epithelium as a substrate.

**Figure 8:**
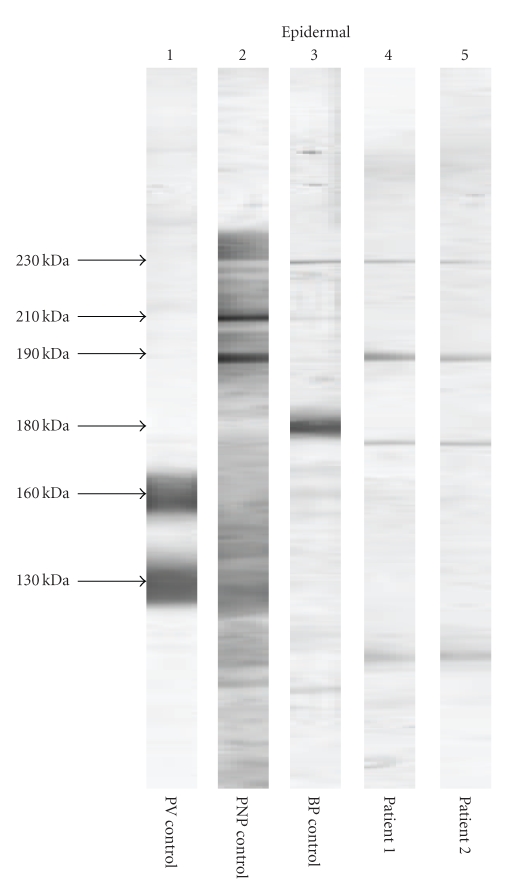
Results of immunoblot analysis using epidermal extract. Patient's sera taken on 2 different occasions (lanes 4 and 5, the time interval was 26 days) recognized BP230 and 190 kDa periplakin, which were also detected by control bullous pemphigoid and control PNP sera. No reaction with 210 kDa envoplakin was observed. Lane 1: pemphigus vulgaris control serum, Lane 2: PNP control serum, and Lane 3: bullous pemphigoid control serum. The serum dilution was 1:20.

**Figure 9:**
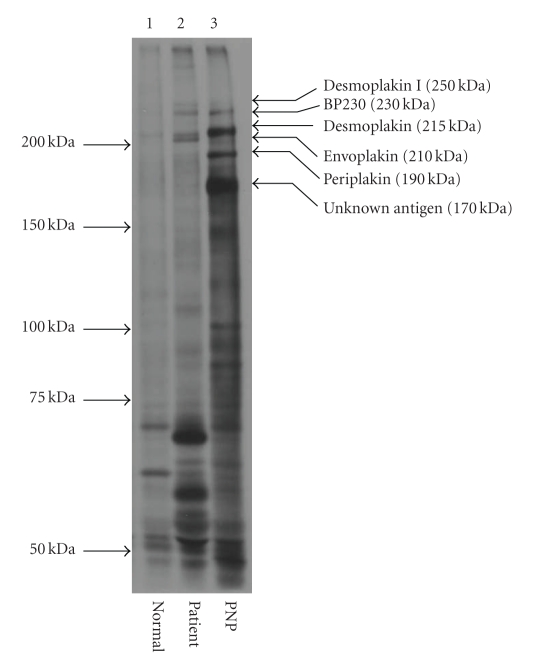
Results of immunoprecipitation using radiolabeled cultured keratinocytes. Control PNP serum (PNP) immunoprecipitated the 250 kDa desmoplakin I, BP230, the 215 kDa desmoplakin II, the 210 kDa envoplakin, the 190 kDa periplakin, and the 170 kDa unknown PNP antigen (lane 3). The serum of this patient (Patient) immunoprecipitated desmoplakin I, BP230 and envoplakin (lane 2). Normal control serum (Normal) showed no positive reaction (lane 1).

 A 49-year-old man presented with a 4-month history of oral lesion and a one-month history of skin lesions on the trunk. He had been admitted to the Department of Internal Medicine for evaluation of an abdominal mass with pain. His past history was unremarkable. On examination, he had blisters and erosions on the buccal mucosa, tongue and lips (Figure 1). The oral lesions did not extend onto the vermillion of the lips. He also had several scattered crusted and erosive lesions with small flaccid bullae on the chest and back (Figure 2). No lichenoid lesions were observed on skin. Biopsy of the flaccid bulla on the chest showed subcorneal bulla formation with mild acantholysis (Figure 3). No interface dermatitis was seen histologically. Histopathological findings of the oral mucosa revealed severe lichenoid dermatitis with dyskeratotic cells (Figure 4). Direct immunofluorescence of the perilesional skin showed intercellular deposits of IgG throughout epidermis and intercellular deposits of C3 in the lower layer of epidermis (Figure 5). Direct immunofluorescence of the oral mucosa showed cell surface deposits of IgG and C3 in the lower layer of epithelium with ovoid bodies of IgG and IgM (Figure 6). Indirect immunofluorescence (IIF) revealed anticell surface antibodies at a titer of 1:160 with normal human skin. IIF was also positive using monkey esophagus and rat bladder epithelium as a substrate (Figure 7) [1]. Anti-Dsg1 antibody index was 46 (positive) and anti-Dsg3 antibody index was 8 (negative) by ELISA using baculovirus protein. Anti-BP230 antibodies and anti-BP180 antibodies were negative by ELISA. By immunoblot analysis using normal human epidermal extract [3], the patient's serum reacted with BP230 and the 190 kDa periplakin, but not with the 210 kDa envoplakin (Figure 8). In contrast, by immunoprecipitation using radiolabeled cultured keratinocytes [1], the serum of this patient immunoprecipitated the 250 kDa desmoplakin I, BP230 and envoplakin, but not the 215 kDa desmoplakin II, periplakin and the 170 kDa unknown PNP antigen (Figure 9).

 A biopsy from the abdominal mass revealed B-cell lymphoma (follicular center cell lymphoma). No pulmonary involvement was seen. We diagnosed the patient with PNP. The patient was transferred to another hospital for the treatment of lymphoma and the clinical course of his eruption thereafter was not known.

## 3. Discussion

 Herein, we present a case of PNP with the clinical and histological features of PF and lichenoid stomatitis in the oral mucosa associated with B-cell lymphoma. This case has the following features: (1) severe mucocutaneous blisters and erosions; (2) histopathological findings of epidermal acantholysis in the skin, dyskeratosis and interface changes in oral mucosa; (3) keratinocyte cell surface deposits of IgG and C3 by direct immunofluorescence; (4) serum autoantibodies that were reactive with normal human skin, monkey esophagus, and rat bladder epithelium; (5) immunoblot demonstration of antibodies to BP230 and the 190 kDa periplakin; (6) immunoprecipitation demonstration of antibodies to desmoplakin I, BP230 and envoplakin; (7) association of B-cell lymphoma. According to these findings, this case fulfilled the criteria of PNP proposed by Anhalt et al. [1, 2], Hashimoto et al. [3], Camisa and Helm [4], and Joly et al. [5], although the reactivity with the 210 kDa envoplakin was negative in immunoblot analysis and the 190 kDa periplakin and the 170 kDa unknown PNP antigen were negative by immunoprecipitation. 

 Initially, the result of IIF using rat bladder epithelium was negative in our case. We tested the IIF again with a different rat bladder epithelium substrate and obtained positive data. Delayed detection of autoantibodies has also been reported in PNP cases [6]. Therefore, it is important to repeat IIF several times using different rat bladder substrate if one result is negative in cases of suspected PNP.

 Amagai et al. reported that all patients with PNP had antibodies to Dsg3 and half of them had antibodies to Dsg1 [7]. They also reported that anti-Dsg3 antibodies could lead to blister formation in neonatal mice and suggested that anti-Dsg3 antibodies are pathogenic in PNP. By contrast, anti-Dsg1 antibodies could not induce blister formation in neonatal mice. In the present case, the patient's sera reacted only with Dsg1, but did not react with Dsg3 by ELISA.

 PNP cases with only anti-Dsg1 antibodies are very rare. There have been only 4 cases reported in the English literature [8–11]. These cases are summarized in Table 1. In 2 of 4 reported cases, anti-Dsg1 antibodies were detected by ELISA as well as by immunoblot analysis and/or immunoprecipitation. In one case, it was detected only by immunoblotting and in another case only by immunoprecipitation. In 3 of 4 reported cases, the clinical features are erythrodermic, wide spread eruption. In one case, the clinical features resembled linear IgA bullous dermatosis. Histologically, acantholysis or cleavage was found at the level of the suprabasal layer in 2 cases and at the level of the upper epidermis in 2 cases. In 3 of 4 cases, lichenoid dermatitis was seen in skin biopsy. All 4 of the cases had oral mucosal erosions despite the absence of anti-Dsg3 antibodies. All the cases had antibodies against the 210 kDa envoplakin and the 190 kDa periplakin detected by immunoprecipitation and/or immunoblot. 

 The pathophysiologic mechanisms of the oral lesion in these PNP cases with only anti-Dsg1 antibodies are unknown. Chorzelski et al. suggested that there may be another unidentified cell surface antigen for PNP, which is related to blister formation in the oral mucous membrane [8]. Fukumoto et al. suggested that other pathological mechanisms, such as interface vacuolar dermatitis, might be involved in the oral mucous membrane lesions in PNP [9]. Unfortunately, no mucosal biopsy was performed in these cases. In our case, mucosal biopsy revealed lichenoid dermatitis. Therefore, we consider that interface dermatitis might contribute to the formation of mucosal lesions in PNP patients with only anti-Dsg1 antibodies. 

 Our present case has several findings that are unique compared with typical PNP cases. First, the skin lesion was very mild with scattered blisters and erosions on the trunk. This clinical feature is also different from most PNP cases with anti-Dsg1 antibodies that exhibit widespread skin eruption. The low titer of autoantibodies and the absence of pathological lichenoid dermatitis may be the reason why the skin eruption was mild in this patient. Histologically, subcorneal acantholytic bulla, typical of PF, was seen in the skin lesion. Therefore, it is convincing to consider that anti-Dsg1 antibodies in this patient are pathogenic and can cause skin eruption. Second, our patient had severe stomatitis, but the oral lesion did not extend to the vermillion border of the lips. In general the characteristic mucosal finding for PNP is severe stomatitis extended to the vermillion border. The absence of anti-Dsg3 antibodies may contribute to the relatively limited oral lesion. Chorzelski et al. also reported a case of PNP with anti-Dsg1 antibodies and observed that the oral mucous membrane involvement was not as severe as in typical cases of PNP [8]. Lastly, no reaction with the 210 kDa envoplakin was seen with immunoblot analysis. We did not know the reason why the 210 kDa envoplakin was not detected. It was able to be detected by immunoprecipitation because immunoprecipitation is a more sensitive test for the detection of antibodies to the plakin family [3].

 Recently Cummins et al. reported 4 cases of PNP without detectable autoantibodies [12]. They concluded that the spectrum of PNP likely to included patients with disease predominantly or exclusively mediated by cytotoxic T cells rather than autoantibodies. Cellular immunity is now likely to be more important in the mechanism of PNP than considered previously. 

 We consider the pathophysiological mechanism of the disease in this patient to be as follows. B-cell lymphoma may dysregulate cellular immunity and cause the oral lichenoid lesion, resulting in damage to basal layer epithelial cells and exposure to self-antigens. Subsequently, autoantibodies to Dsg1 are formed, which produce the PF lesion on the trunk. Based on the clinical and histological findings, we consider that the skin lesion was produced by humoral immunity, whereas the oral lesion was produced by cellular immunity.

## Figures and Tables

**Table 1:** PNP cases with anti-Dsg1 antibodies but not with anti-Dsg3 antibodies.

No	References	Age/Sex	Skin lesion	Oral lesion	IP	IB	ELISA
1	Chorzelski et al. [8]	80/F	widespread erythema, bullae, erosions	bullae and erosions	250 kD	230 kD	Dsg1: 190.6 (+) Dsg3: 4.1 (−)
					230 kD	210 kD	
					210 kD	190 kD	
					190 kD	160 kD	

2	Martel et al. [11]	70/M	generalized lichenoid, exfoliative erythroderma	erosions	NM	250 kD	NM
						210 kD	
						190 kD	
						160 kD	

3	Lee et al. [10]	69/M	annular erythema, with bulla on extremities	erosions	210 kD	NM	NM
					190 kD		
					160 kD		

4	Fukumoto et al. [9]	64/F	erythrodermic lichenoid dermatitis and bullae	erosions	NM	210 kD	Dsg1: 70 Dsg3: (−)
						190 kD	

5	Present case	49/M	scattered bullae and erosions on trunk	bullae and erosions	250 kD	230 kD	Dsg1: 46 (+) Dsg3: 8 (−)
					230 kD	190 kD	
					210 kD		

IP: immunoprecipitation, IB: immunoblotting, NM: not mentioned.
